# *Codonopsis lanceolata* Extract Prevents Diet-Induced Obesity in C57BL/6 Mice

**DOI:** 10.3390/nu6114663

**Published:** 2014-10-28

**Authors:** Jong Seok Lee, Kui-Jin Kim, Young-Hyun Kim, Dan-Bi Kim, Gi-Hae Shin, Ju-Hyun Cho, Bong Kyun Kim, Boo-Yong Lee, Ok-Hwan Lee

**Affiliations:** 1 Department of Food Science and Biotechnology, Kangwon National University, Chuncheon 200-701, Korea; E-Mails: jongseoklee78@gmail.com (J.S.L.); vvkyh@naver.com (Y.-H.K.); danbekim22@nate.com (D.-B.K.); cordelia162@hanmail.net (G.-H.S.); 2 Department of Nutrition and Food Science, College of Agriculture and Natural Resources, University of Maryland, College Park, MD 20742, USA; E-Mail: kjkim@umd.edu; 3 Hurum Central Research Institute, Ochang 363-883, Korea; E-Mails: dusvnd608@hurum.co.kr (J.-H.C.); kp2307@hurum.co.kr (B.K.K.); 4 Department of Food Science and Biotechnology, CHA University, Seongnam 463-836, Korea; E-Mail: bylee@cha.ac.kr

**Keywords:** obesity, *Codonopsis lanceolata*, body weight, cholesterol, triglyceride

## Abstract

*Codonopsis lanceolata* extract (CLE) has been used in traditional medicine in the Asian-Pacific region for the treatment of bronchitis, cough, and inflammation. However, it is still unclear whether obesity in mice can be altered by diet supplementation with CLE. To investigate whether CLE could have preventative effects on high fat diet (HFD)-induced obesity, male C57BL/6 mice were placed on either a normal chow diet, 60% HFD, or a HFD supplemented with CLE (60, 180, and 360 mg/kg/day) for 12 weeks. CLE decreased body weight and subcutaneous and visceral fat weights in HFD-induced obese mice. CLE group mice showed lower fat accumulation and a smaller adipocyte area in the adipose tissue compared with the HFD group mice. CLE group mice exhibited lower serum levels of triglycerides, total cholesterol, low density lipoprotein (LDL), glucose, and insulin compared with the HFD group mice. In addition, CLE decreased liver weight and lowered the increase in aspartate aminotransferase (AST) and alanine transaminase (ALT) levels in HFD-induced obese mice. These results indicate that CLE can inhibit the development of diet-induced obesity and hyperlipidemia in C57BL/6 mice.

## 1. Introduction

Obesity has recently emerged as a major health problem and risk factor for various disorders worldwide [1,2]. A high-calorie diet is the one of the major factors contributing to the development of obesity [3]. Specifically, it has been reported that administration of a high fat diet (HFD) results in abnormal lipid accumulation in the liver [4] and an increase in triglyceride, total cholesterol, and low density lipoprotein (LDL) in the blood serum [5]. It is very difficult to achieve weight loss and improve its abnormality once weight exceeds the normal range. Therefore, the prevention of obesity and effective intervention to reduce obesity are of great importance for public health [6]. Current obesity treatments involve diet and exercise, which rarely meet the patient needs, and additional pharmacotherapy is often required. Reductil, also known as sibutramine, is approved for long-term use with diet and exercise as an appetite suppressant for the treatment of obesity; however, patients often experience eventual weight regain and side effects [7,8]. This tendency has led to an increase in the consumption of functional foods for obesity prevention, leading to a strong demand for the development of new sources of functional foods and related studies on their efficacy [9,10,11]. *Codonopsis lanceolata* has been used as a complementary herbal medicine as an anti-oxidant, anti-inflammation, anti-adipogenesis, and anti-cancer agent in the Asia-Pacific region [12,13,14]. Recently, it has been reported that *C. lanceolata* regulates HFD-induced obesity in rats [15] and also improves alcoholic-induced hepatic steatosis in rats [16].

It is difficult to ascertain the exact mechanisms for the long-term progression of obesity in humans. To circumvent some of these issues, the animal model of diet-induced obesity has become one of the most important tools for understanding the development of obesity [17]. There have been many studies characterizing the responses of animals exposed to HFD. Some animals show profound increases in their body fat content, while some are resistant to weight gain with HFD [18]. Among these animals, the outbred Spraque-Dawley rats have different responses with regard to the development of obesity when fed with HFD. The A/J mouse and C57BL/KsJ mouse are relatively resistant to HFD compared to the C57BL/6 mouse [19]. The metabolic abnormalities of the C57BL/6 mouse closely parallel that of human obesity progression because when fed with a HFD, these mice develop obesity, hyperinsulinemia, and hyperglycemia [20]. However, a protective effect of *C. lanceolata* extract against obesity progression in C57BL/6 mice fed with HFD has yet to be confirmed. Therefore, the current study was performed to determine whether dietary supplementation with *C. lanceolata* root extract (CLE) attenuates the development of obesity in C57BL/6 mice fed a HFD.

## 2. Materials and Methods

### 2.1. Materials

*Codonopsis lanceolata* root harvested in 2012 was purchased from the Jeongseon Market (Gangwon, Korea). The extract of *C. lanceolata* was obtained as follows. Briefly, *C. lanceolata* root (100 g) was extracted with distilled water (2 L) at 90 °C for 4 h using a shaking extraction method and then allowed to cool at room temperature. After the extract was centrifuged, the supernatants were filtered with Whatman filter paper (0.2 µm) and concentrated to 15 Brix using a vacuum rotary evaporator (Eyela, Tokyo, Japan) at 50 °C. Then, the extract was freeze-dried using a freeze dryer (Ilshin, Seoul, Korea). CLE was dissolved in distilled water for oral administration. The phenolic compounds in the samples were analyzed using high performance liquid chromatography with diode-array detection HPLC-DAD analyses, which were performed on an Agilent series 1100 HPLC (Arcade, NY, USA) instrument allowing the determination at different wavelengths (280, 320, and 370 nm) of single molecules belonging to different subclasses [21]. In detail, the Nucleosil 100-5 C-18 column (250 mm × 4.0 mm i.d., 5 μm particle size) was used, which was protected by a 10 mm guard column. The eluents were water at pH 3.29 by formic acid (0.035%, v/v) (A) and acetonitrile (B). The analyses were performed by a multistep linear solvent gradient as follows: 0%–15% B (45 min), 15%–30% B (15 min), 30%–50% B (5 min), 50%–100% B (5 min) and 100%–0% B (10 min). The diode array detector was monitored at 270 nm, and the injection volume of the samples was 10 µL. The standards used for the analysis were 4-hydroxyl benzohydrazide, gallic acid, vanillic acid, *p*-anisic acid, alizarin, chlorogenic acid, caffeic acid, syringic acid, *p*-coumaric acid, *trans*-ferulic acid, catechin, epigallocatechin gallate, quercetin hydrate, myricetin, morin hydrate, 3-hydroxyflavone, rutin, and naringin, all of which were purchased from Sigma (St. Louis, MO, USA). The results are shown in Table 1.

**Table 1:** The major phytoconstituents composing the aqueous extracts of *C. lanceolata*.

Compounds	Contents (mg/100 g Extract)
*Phenolic acid*	
Phloroglucinol	924.58
Gallic acid	85.28
Chlorogenic acid	32.99
Caffeic acid	37.18
*p*-Coumaric acid	2.23
*Trans*-ferulic acid	16.40
Total of phenolic acid	1098.66
*Flavonoids*	
(−)-Epigallocatechin	1353.00
Epigallocatechin gallate	20.64
Rutin hydrate	391.08
Luteolin	12.12
Total of flavonoids	1776.84

### 2.2. Anti-Obesity Activity of Codonopsis lanceolata Root Extract

#### 2.2.1. Study Design

Sixty male C57BL/6 mice (8 weeks old) were purchased from Jackson Laboratories (Bar Harbor, ME, USA) and used after 2 weeks of quarantine and acclimation. The animals were kept in the animal facility of the Deajeon University in a light-controlled room (12-h light/dark cycle) at an average temperature of 25 ± 2 °C and relative humidity of 50% ± 5%. This experiment was conducted in facilities approved by the Institutional Animal Care and Use Committee (IACUC) of the Deajeon University (Approval NO.: DJUARB2012-013; approval date: 30 August 2012). All mice had free access to food and water. They were fed an AIN-76A-based diet supplemented with or without 60 kcal% fat (Research diet, New Brunswick, NJ, USA) throughout the 12-week treatment period. The animals were subdivided into six groups: (1) normal chow diet (ND) group, *n* = 10; (2) HFD + normal saline group, *n* = 10; (3) HFD + reductil (CBT, 2 mg/kg) group, *n* = 10; (4) HFD + CLE (60 mg/kg BW/day), *n* = 10; (5) HFD + CLE (180 mg/kg BW/day), *n* = 10; (6) HFD + CLE (360 mg/kg BW/day), *n* = 10. The sample size for this current study was selected based on the effective size observed in experiments conducted previously with similar design [22], with a desired power of 0.8 and an alpha level of 0.05 using SigmaPlot software (Systat Software Inc., Richmond, CA, USA). The calculated sample size with two different groups (HFD/ND) per substance was 10 animals per group. CLE and reductil were orally administered to the mice by gavage every day throughout the 12-week treatment period. Body weights were measured at the initiation of treatment, every week thereafter, and on the day of sacrifice. Food intake and water consumption were measured in mg/kg body weight (BW)/day at the start of treatment and at weekly intervals thereafter. The amount of food and water was measured before being supplied to the cage, and the remainder was measured the next day.

#### 2.2.2. Blood Chemistry

At the end of the treatment, all mice were fasted for 12 h, anesthetized with isoflurane and euthanized; arteriovenous blood was collected. The collected blood was processed using a microcentrifuge method, and the serum was stored in a freezer at −70 °C. The blood samples were evaluated for serum lipid, glucose, and insulin levels. The concentrations of total cholesterol (TC), triglyceride (TG), high density lipoprotein (HDL) cholesterol, and LDL cholesterol were determined using a commercial kit (Asan Pharmaceutical Co., Seoul, Korea) based on the enzymatic colorimetric method. The level of LDL cholesterol was determined using the Friedewald formula, where

LDL cholesterol = TC − HDL cholesterol − (TG/5). (1)

#### 2.2.3. Specimen Collection

After collection of blood, subcutaneous and visceral adipose and liver tissues were removed, rinsed with phosphate buffered saline, and weighed.

#### 2.2.4. Determination of Fat Size of Adipose Tissue by Oil Red O Staining

Adipose tissues were stained with Oil Red O, an indicator of tissue lipid content, as described in [23] with slight modifications. Briefly, adipose tissue was washed with phosphate-buffered saline, fixed with 10% neutral buffered formalin and stained with Oil Red O solution (0.5 g in 100 mL isopropanol) for 10 min. The Oil red O was purchased from Sigma (St. Louis, MO, USA).

#### 2.2.5. Histology Preparation and Hematoxylin and Eosin Stain (H & E)

Organs were fixed and preserved in 10% neutral phosphate buffered formalin. Formalin-fixed liver tissues were embedded in paraffin using standard procedures. Sections (5 µm thick) were cut and stained with hematoxylin and eosin (H & E) [24]. Collected tissues were grossly and microscopically examined.

### 2.3. Statistical Analysis

The results are presented as means ± SEM. The data were analyzed using ANOVA and Duncan’s multiple range tests. A level of *p* < 0.05 was regarded as statistically significant.

## 3. Results

### 3.1. CLE Suppresses Body Weight Gain and Body Fat Accumulation in C57BL/6 Mice Fed HFD

**Figure 1:**
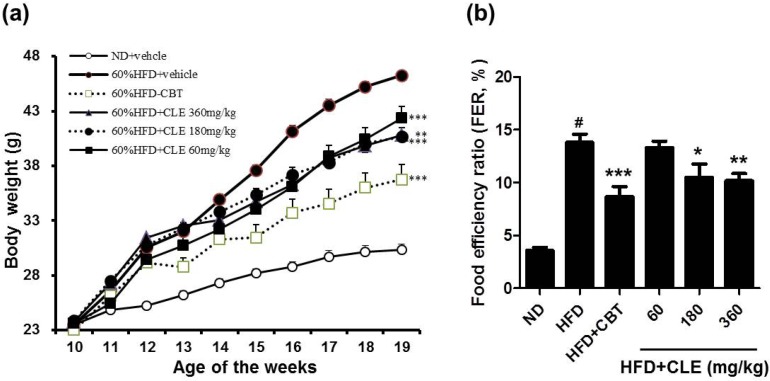
Effect of *Codonopsis lanceolata* extract (CLE) on body weight and food efficiency ratio (FER) in high fat diet (HFD)-fed mice. (**a**) Effect of CLE on body weight gain in HFD-fed mice. (**b**) Effect of CLE on FER in HFD-fed mice. Food efficiency ratio (FER) = increased body weight (g)/food intake (g). Values are the mean ± SEM (*n* = 10), # *p* < 0.05 *vs.* ND-fed mice, * *p* < 0.05, ** *p* < 0.01, *** *p* < 0.001 *vs.* HFD-fed mice. ND: normal chow diet, HFD: 60% high fat diet + vehicle, HFD + CBT: 60% high fat diet + reductil (CBT, 2 mg/kg), HFD + CLE: 60% high fat diet + CLE (60, 180 and 360 mg/kg, respectively).

To evaluate the preventative effects of CLE on HFD-induced obesity, we investigated the development of HFD-induced obesity in C57BL/6 mice with and without CLE supplementation for 12 weeks. As shown in Figure 1, mice fed HFD increased their body weight and food efficiency after 12 weeks of feeding compared with mice fed a ND. According to the food efficiency ratio (FER) equation, a change in body weight is the most important factor affecting the FER, as there is no large change in the amount of ingested food. Thus, it is possible to apply the FER as an indicator; a small value for the FER can effectively predict the avoidance of obesity.

**Figure 2:**
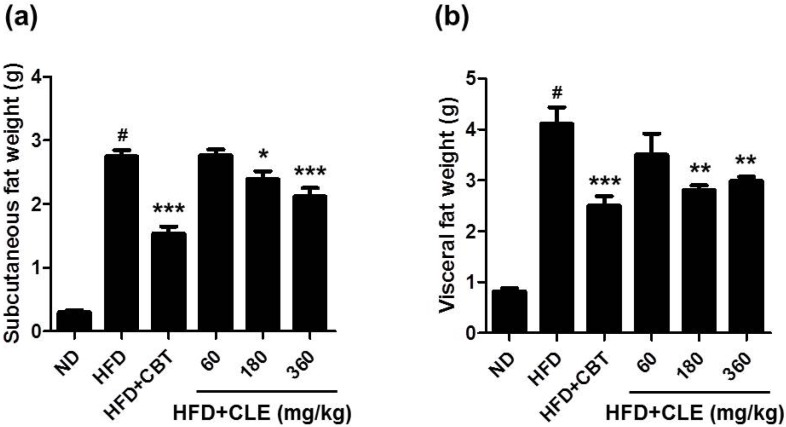
Effect of CLE on adipose tissue weights in HFD-fed mice. (**a**) Effect of CLE on subcutaneous fat weight in HFD-fed mice. (**b**) Effect of CLE on visceral fat weight in HFD-fed mice. Values are the mean ± SEM (*n* = 10), # *p* < 0.05 *vs.* ND-fed mice, * *p* < 0.05, ** *p* < 0.01, *** *p* < 0.001 *vs.* HFD-fed mice. ND: normal chow diet, HFD: 60% high fat diet + vehicle, HFD + CBT: 60% high fat diet + reductil (CBT, 2 mg/kg), HFD + CLE: 60% high fat diet + CLE (60, 180 and 360 mg/kg, respectively).

**Figure 3:**
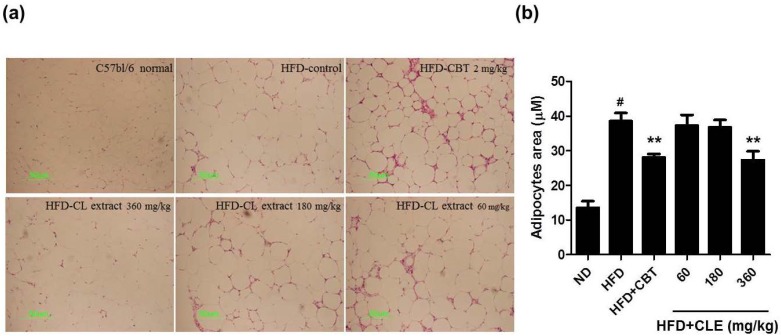
Effect of CLE on adipocyte area in adipose tissue of mice fed a HFD. (**a**) Representative sections of adipose tissue in different groups. Lipid accumulation was visualized using Oil Red O staining. (**b**) Morphometric analysis of adipose tissue was performed using MetaMorph. Adipocyte area was quantified for each genotype and values represent mean ± SEM (*n* = 10), # *p* < 0.05 *vs.* ND-fed mice, ** *p* < 0.01 *vs.* HFD-fed mice. ND: normal chow diet, HFD: 60% high fat diet + vehicle, HFD + CBT: 60% high fat diet + reductil (CBT, 2 mg/kg), HFD+CLE: 60% high fat diet + CLE (60, 180 and 360 mg/kg, respectively).

Mice that were orally administered CLE (60, 180, and 360 mg/kg) with HFD showed decreased body weight and food efficiency compared to HFD-fed mice. Body weights were suppressed by 2.6%, 25.9%, and 26.1% when treated with 60, 180, and 360 mg/kg CLE compared to the weights of the HFD control group. The results of body weight and FER indicated that obesity was reduced upon administration of CLE. Mice that were orally administered CLE with HFD had a lower amount of both subcutaneous and visceral fat compared to the HFD control group (Figure 2). In addition, histological analysis showed a higher concentration of disrupted fat accumulation in the adipose tissue of the CLE treated group compared than in the HFD control group (Figure 3). These results suggest that the HFD resulted in obesity, but the administration of CLE suppressed body weight gain and excess fat accumulation in adipose tissue in HFD-fed C57BL/6 mice.

### 3.2. CLE Ameliorate Impaired Lipid, Glucose, and Insulin Homeostasis in HFD-Induced Obese Mice

**Figure 4:**
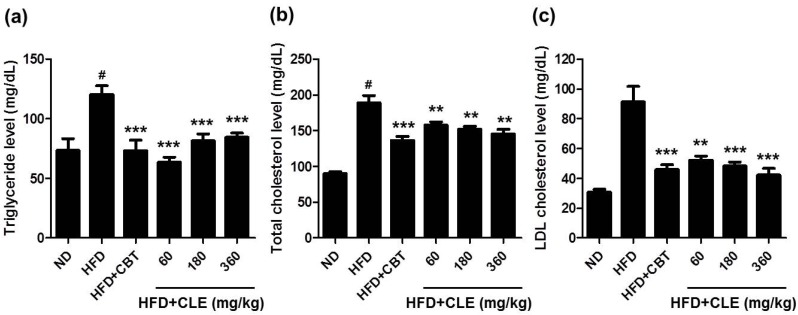
Effect of CLE on serum cholesterol levels of mice fed a HFD. (**a**) Serum triglyceride. (**b**) Serum total cholesterol. (**c**) Serum LDL cholesterol. The serum biochemical parameters were assessed in mice fed a ND, a HFD, a HFD with reductil (2 mg/kg), and a HFD with CLE (60, 180 and 360 mg/kg). Values are the mean ± SEM (*n* = 10), # *p* < 0.05 *vs.* ND-fed mice, ** *p* < 0.01, *** *p* < 0.001 *vs.* HFD-fed mice.

**Figure 5:**
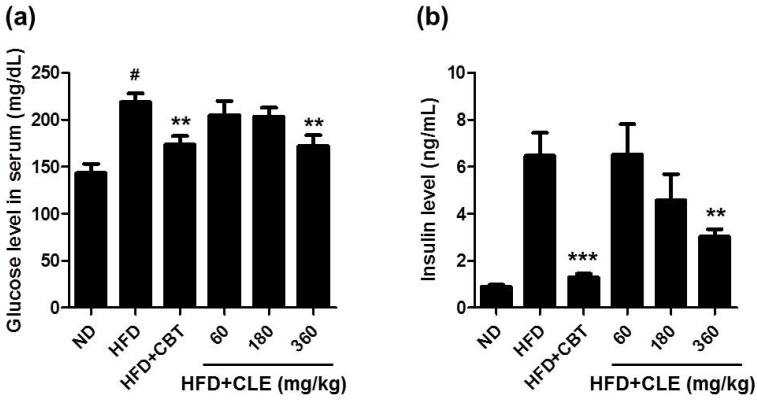
Effect of CLE on serum glucose and insulin levels of mice fed a HFD. (**a**) Serum glucose. (**b**) Serum insulin. Values are the mean ± SEM (*n* = 10), # *p* < 0.05 *vs.* ND-fed mice, ** *p* < 0.01, *** *p* < 0.001 *vs.* HFD-fed mice. ND: normal chow diet, HFD: 60% high fat diet + vehicle, HFD + CBT: 60% high fat diet + reductil (CBT, 2 mg/kg), HFD + CLE: 60% high fat diet + CLE (60, 180 and 360 mg/kg, respectively).

The metabolic abnormalities of the C57BL/6 mouse closely parallel those of human obesity because when fed *ad libitum* with a HFD, these mice develop obesity, which has been associated with dyslipidemia, hyperglycemia, and hyperinsulinemia [25,26]. To evaluate the effects of CLE on serum lipid profiles, glucose, and insulin, we examined various systemic parameters in the C57BL/6 mice fed HFD with or without CLE. As expected, the HFD control group mice had higher serum levels of TG, TC, LDL, glucose, and insulin than the ND group (Figure 4 and Figure 5). These results indicated that HFD caused hypercholesterolemia, hyperglycemia, and hyperinsulinemia. Compared with the HFD control group mice, the CLE-treated group mice showed a significant decrease in these serum metabolic parameters (Figure 4 and Figure 5). These results demonstrate that CLE-treated mice have a recovering trend compared to HFD-induced obese mice, suggesting that CLE treatment produces the improved lipid, glucose, and insulin homeostasis found in HFD-induced obese mice.

### 3.3. CLE Suppresses Hepatic Steatosis in HFD-Induced Obese Mice

It has been reported that hepatic steatosis is commonly associated with obesity [27]. Increases in blood transaminase activities such as serum aspartate aminotransferase (AST) and serum alanine aminotransferase (ALT) in obese patients are caused by fatty liver development. Especially the development of fatty liver seems to elevate ALT in the blood [28].

**Figure 6:**
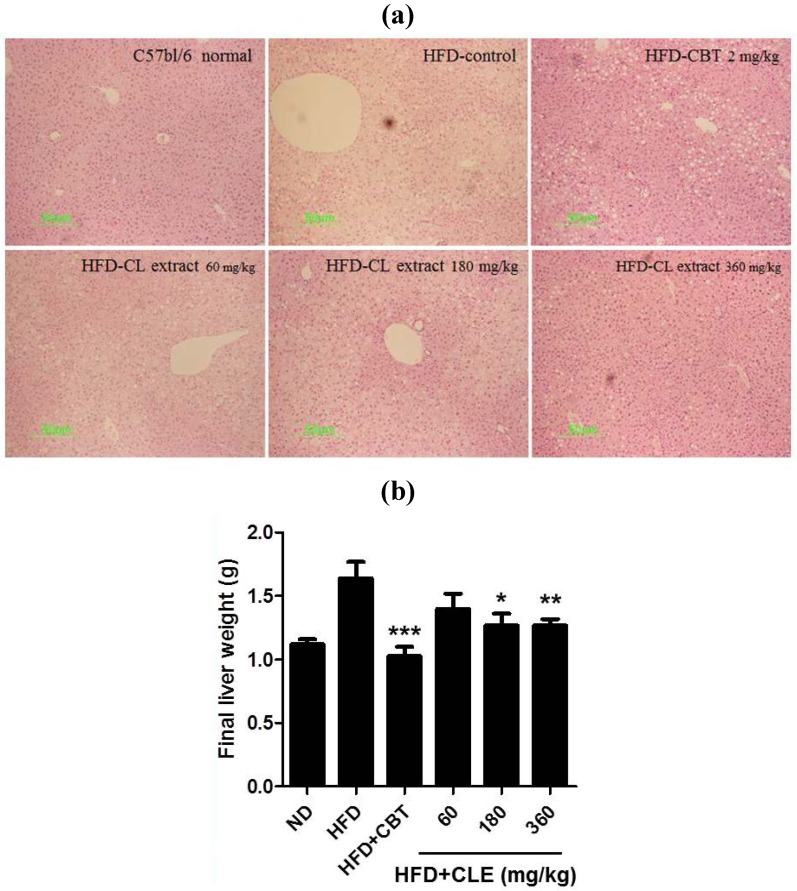
Effect of CLE on hepatic steatosis in HFD-fed mice. (**a**) Representative sections of liver tissue stained with hematoxylin and eosin (H & E). (**b**) Effect of CLE on liver weight in HFD-fed mice. Values are the mean ± SEM (*n* = 10), * *p* < 0.05, ** *p* < 0.01, *** *p* < 0.001 *vs.* HFD-fed mice.

**Figure 7:**
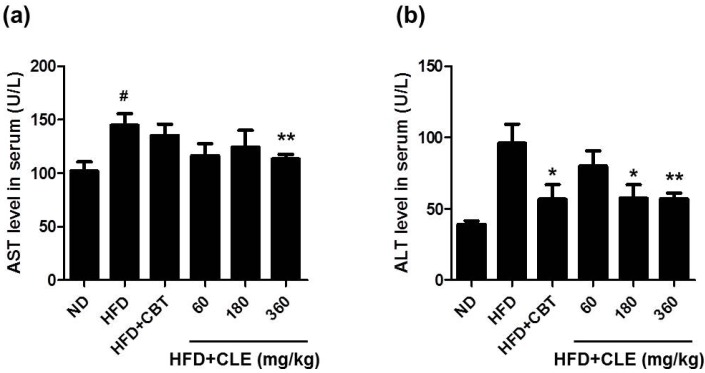
Effect of CLE on serum AST and ALT levels of mice fed a HFD. (**a**) Serum AST. (**b**) Serum ALT. Values are the mean ± SEM (*n* = 10), # *p* < 0.05 *vs.* ND-fed mice, * *p* < 0.05, ** *p* < 0.01 *vs.* HFD-fed mice. ND: normal chow diet, HFD: 60% high fat diet + vehicle, HFD + CBT: 60% high fat diet + reductil (CBT, 2 mg/kg), HFD + CLE: 60% high fat diet + CLE (60, 180, and 360 mg/kg, respectively).

Therefore, we performed histopathological analysis of the liver to determine whether CLE could affect hepatic steatosis in HFD-induced obese mice. As shown in Figure 6, HFD group mice exhibited increased liver weight (Figure 6B) and severe hepatic steatosis by both gross morphological examination and histological examination, with the latter showing the liver as having hepatic vacuoles, and lipid droplets (Figure 6A). In addition, liver injury was verified by highly elevated levels of serum AST and ALT (Figure 7A,B). These results indicated that HFD caused hepatic steatosis and liver injury in mice. CLE decreased liver weight in HFD-induced obese mice. From the results of histological analysis, CLE treatment has a hepatoprotective effect on HFD-induced steatosis (Figure 6). CLE treatment also protected against and lowered the increase in ALT levels found in the HFD-fed mice (Figure 7). These results indicate that CLE confers protection against hepatic steatosis in HFD-induced obese mice.

## 4. Discussion

Obesity is reaching pandemic proportions throughout the world. As obesity is a complex and chronic disease, it is difficult ascertain the exact mechanisms for this long-term process in humans [2]. Fat-rich foods have been shown to produce increased body weight and diabetes in various strains of mice and rats [17]. Among these animals, the C57BL/6 mouse is a particularly good experimental model mimicking the progression of human obesity [20]. In recent years, it was demonstrated that CLE could regulate HFD-induced obesity in rats [15] and could improve alcoholic-induced hepatic steatosis [16]. However, anti-obesity effects of CLE had never been investigated in C57BL/6 mice fed HFD. In this study, we investigated the effects of CLE on the development of HFD-induced obesity in C57BL/6 mice. When C57BL/6 mice were fed a HFD, these mice developed obesity, hyperglycemia, and hyperinsulinemia, but when fed a normal chow diet, they remained lean without metabolic abnormalities. Mice administered CLE showed significant differences in body weight changes, fat accumulation, and serum glucose and insulin levels compared with HDF control. Our results show that CLE treatment is sufficient to attenuate HFD-induced obesity, hyperglycemia, and hyperinsulinemia in C57BL/6 mice [15].

Plants have been known to have the ability to synthesize a wide variety of chemical compounds with important beneficial effects [29]. Many studies have demonstrated that plant-derived active components have a number of health benefits [30,31]. The root of *C. lanceolata* is composed of various active components including tannins, saponins, polyphenols, alkaloids, essential oils, and steroids [32,33]. In the present study, we found that CLE contained various active components such as epigallocatechin, phloroglucinol, gallic acid, chlorogenic acid, and epigallocatechin gallate. Inhibition of adipocyte differentiation has been suggested as an important strategy for preventing or treating obesity. It was reported that phloroglucinol inhibited adipocyte differentiation and lipid accumulation in 3T3-L1 via inhibition of the expression levels of several adipocyte maker genes, including proliferator activated receptor γ (PPARγ) and CCAAT/enhancer-binding protein α (C/EBPα) [34]. Chlorogenic acid improves the disordered glucose/lipid metabolism in db/db mice by modulating the secretion of adipokines, upregulating hepatic PPAR-α, and inhibiting glucose-6-phosphatase [35]. Epigallocatechin and epigallocatechin gallate have been demonstrated in cell cultures and animal models of obesity to reduce adipocyte differentiation and proliferation, body weight, fat mass, plasma levels of TG, free fatty acids, cholesterol, glucose, and insulin, as well as to increase fat oxidation [36,37,38]. Gallic acid protects against hepatic steatosis and insulin resistance in HFD-fed nonalcoholic C57BL/6 mice with fatty liver disease [22].

Obesity causes abdominal visceral fat and weight gain due to HFD [5,39]. Accumulation of fat in the abdominal viscera is associated with serum TG, TC, and LDL, which are defined as the main risk factors for dyslipidemia. Higher levels of serum TG have been implicated in cardiovascular disease [40]. TC and LDL are also associated with the occurrence of coronary heart disease [41,42]. Therefore, regulation of blood TG, TC, and LDL is important for maintaining homeostasis in the body. In the present study, HFD-fed mice showed more serum TG, TC, and LDL levels compared with those of ND-fed mice. We found that CLE suppressed TG, TC, and LDL levels in the blood harvested from C57BL/6 mice fed HFD. HFD-induced obesity and abnormal lipid metabolism are associated with nonalcoholic fatty liver disease leading to hepatic failure and causing a boost in serum glutamic oxaloacetic transaminase (SGOT) and serum glutamate pyruvate transaminase (SGPT) levels in the serum [43]. The effect of increased liver enzyme levels and the formation of hepatic steatosis in the HDF-fed group are associated with a significant increase in liver weight [44]. HFD group mice exhibited increased total liver weight. Liver injury was also confirmed by significant increases of serum AST and ALT in the HFD-fed group. An elevated serum activity of ALT has been suggested as the fatty liver disease in the general human population [45]. We found that CLE-treated mice showed attenuated liver weight and serum AST and ALT levels compared to those of the HFD-fed group. These findings showed that the CLE treatment had a significant hepatoprotective effect in HFD-induced obese mice.

## 5. Conclusions

*Codonopsis lanceolata* has been widely used as a folk medicine in Korea and has been found to contain polyphenolic compounds, which have been implicated in the prevention and treatment of obesity. We found that C57BL/6 mice fed with HFD for 12 weeks increased in body weight and adipose tissue weight, accompanied by fatty liver, hyperglycemia, hyperinsulinemia, hypercholesterolemia, and high levels of serum AST and ALT as opposed to the normal control ND group. The CLE-treated groups showed a significant decrease in body weight. Furthermore, CLE supplementation attenuated adipose tissue weight and reduced serum TG, TC, glucose, and insulin levels in HDF-fed mice. Altogether, CLE had marked effects on inhibiting the development of obesity and hyperlipidemia in obese C57BL/6 mice fed HFD. These findings suggest that *Codonopsis lanceolata* as a complementary herbal medicine may attenuate some of the physiological changes that occur in obesity induced by HFD.
